# The quick sequential organ failure assessment score as a predictor of mortality in septic dogs

**DOI:** 10.18849/ve.v10i4.723

**Published:** 2025-10-06

**Authors:** Jacqueline Chappell+

**Keywords:** CANINE, DOG, MORTALITY, PROGNOSIS, SEPSIS, QSOFA

## Abstract

**PICO question:**

In adult dogs with suspected or confirmed sepsis, does the quick Sequential Organ Failure Assessment (qSOFA) score accurately predict mortality?

**Clinical bottom line:**

**Category of research:**

Prognosis.

**Number and type of study designs reviewed:**

Four papers were critically reviewed. All four were retrospective cohort studies.

**Strength of evidence:**

Weak.

**Outcomes reported:**

The use of the quick Sequential Organ Failure Assessment (qSOFA) score to predict mortality of septic dogs in a veterinary hospital setting produced mixed results. When applied generally to dogs admitted to the Intensive Care Unit (ICU) in the first study, there was no significant difference in score between survivors and non-survivors, and this pattern was consistent among the septic dogs in that study. The qSOFA was also not found to be predictive of mortality in dogs with severe sepsis and septic shock in the second study. However, when applied to female dogs referred to a tertiary hospital for pyometra in the third study and dogs with a clinical diagnosis of sepsis requiring surgical source control in the fourth, odds of mortality were higher for scores more than or equal to 2, and discrimination between survivors and non-survivors was good.

**Conclusion:**

The available literature does not definitively validate the use of qSOFA as a standalone prognostic tool for dogs with sepsis. It may serve as a supplementary tool for early identification of at-risk patients; however, it should not replace clinician judgment in decision-making. As is the case when applied to humans in the literature, the score may be used in conjunction with other observations to aid in clinical decision-making, but a negative result should not delay investigations or treatments. A higher score or an increase in score may be used to identify patients at risk of deterioration to trigger further investigations and consideration of possible infection. The qSOFA should only be used as a complementary tool to assist with management and monitoring, not to determine prognosis for individual patients.

## How to apply this evidence in practice

The application of evidence into practice should take into account multiple factors, not limited to: individual clinical expertise, patient’s circumstances and owners’ values, country, location or clinic where you work, the individual case in front of you, the availability of therapies and resources.

Knowledge Summaries are a resource to help reinforce or inform decision-making. They do not override the responsibility or judgement of the practitioner to do what is best for the animal in their care.

## Clinical scenario

You are a small animal veterinarian working at a referral hospital. Concerned about identifying dogs with confirmed or suspected sepsis who are at high risk of mortality, you decide to investigate the quick Sequential Organ Failure Assessment (qSOFA) to facilitate earlier interventions and improve patient outcomes.

## The evidence

The quick Sequential Organ Failure Assessment (qSOFA) score has been validated in people for identifying individuals with a suspected infection at high risk of mortality (Singer et al., 2016). Sepsis is a life-threatening syndrome characterised by a dysregulated host response to infection causing organ dysfunction (Singer et al., 2016). Patients are at higher risk for poor outcomes if they meet at least 2 of the qSOFA criteria: respiratory rate more than or equal to 22/minute, systolic blood pressure less than or equal to 100 mmHg or altered mentation (Singer et al. 2016). This tool has recently been explored in canine practice. Four retrospective cohort studies assessed the efficacy of qSOFA as a prognostic tool in a veterinary hospital setting (Ortolani & Bellis, 2021; Stastny et al., 2021; Osgood et al., 2022; Donati et al., 2022). One study included dogs admitted to the Intensive Care Unit (ICU), 14 of which were septic (Ortolani & Bellis, 2021) and another only included dogs with pyometra (Donati et al., 2022). The remaining two required a clinical diagnosis of sepsis, defined as meeting more than or equal to 2 Systemic Inflammatory Response Syndrome (SIRS) criteria and evidence of infection (Stastny et al, 2021; Osgood et al., 2022). Osgood et al. (2022) focused on dogs with severe sepsis and septic shock, requiring evidence of organ dysfunction and Stastny et al. (2021) required that surgical source control was pursued. Though dogs with non-septic conditions were evaluated in Ortolani & Bellis (2021), this Knowledge Summary will focus on the evidence related to dogs with sepsis. All four studies measured survival to discharge and non-survival either by death or euthanasia. Ortolani & Bellis (2021) and Osgood et al. (2022) found no significant differences in qSOFA score between survivors and non-survivors, whereas Donati et al. (2022) and Stastny et al. (2021) found that qSOFA scores more than or equal to 2 were significantly associated with mortality. The outcomes are contradictory despite similarities in study design and recruitment of cases. The evidence was of weak strength across the board – all studies were retrospective and influenced by many of the same potential confounding factors. As such, the available evidence does not definitively confirm nor disqualify the use of qSOFA as a prognostic tool. More work is required to evaluate its utility as a patient assessment instrument.

## Summary of the evidence

**Donati et al. (2022):** Retrospective evaluation of the use of quick Sepsis-related Organ Failure Assessment (qSOFA) as predictor of mortality and length of hospitalization in dogs with pyometra (2013–2019): 52 cases **Aim: **To assess the prognostic value of the qSOFA score for predicting in-hospital mortality and length of stay in dogs diagnosed with pyometra.

Population:	Female canine patients referred to a tertiary hospital for treatment of pyometra between February 2013 and April 2019. The age range was not specified.
Sample size:	52 dogs.
Intervention details:	Patients were assigned a quick Sequential Organ Failure Assessment (qSOFA) score between 0 and 3: respiratory rate (RR) ≥ 22/minute = 1, altered mentation Acute Patient Physiologic and Laboratory Evaluation (APPLE) 0 (normal) = 0 and 1–4 (abnormal) = 1, systolic blood pressure (SBP) (measured with a Doppler) ≤ 100 mmHg = 1. A score of 0 was assigned if these were within normal limits. Dogs were also classified as Systemic Inflammatory Response Syndrome (SIRS) 1–3 based on the presence of the following abnormal parameters: SIRS 1 = ≥2 of: temperature °C (< 38.1 or > 39.2), heart rate (> 120 bpm), white blood cell count (< 6 or > 16.6 x 10^^9^/L). SIRS 2 = ≥ 2 of: temperature °C (< 37.2 or > 39.4), heart rate (> 150 bpm), white blood cell count (< 5 or > 19.9 x 10^^9^/L). SIRS 3 = 3 of: temperature °C (< 37.2 or > 39.4), heart rate (> 150 bpm), white blood cell count (< 5 or > 19.9 x 10^^9^/L) .
Study design:	Retrospective cohort study.
Outcome Studied:	Survival or non-survival of the patient.
Main Findings (relevant to PICO question):	Odds of mortality was 6.5 times higher for patients with a qSOFA ≥ 2 (25 dogs) than in patients with a qSOFA score < 2 (27 dogs) (P = 0.019, 95% CI 1.4–31.3). Area Under the Receiver Operating Characteristic (AUROC) curve = 0.72 (95% CI 0.59–0.85), with a sensitivity of 77.8%, and specificity of 66.7%. Overall survival 34/52 (65%). Of the 18 non-survivors, 7 were euthanised for poor response to treatment or disease progression.
Limitations:	Retrospective study. Only included dogs with pyometra (confirmed with exploratory laparotomy) no other sources of infection. Human cut-off values used for RR and SBP. SIRS criteria timing from 48 hours before and 24 hours after onset of infection rather than at presentation. Not compared to other illness severity scores (eg Sequential Organ Failure Assessment (SOFA), Acute Patient Physiologic and Laboratory Evaluation (APPLE) to evaluate performance. No control population.

**Ortolani & Bellis (2021):** Evaluation of the quick sequential organ failure assessment score plus lactate in critically ill dogs **Aim: **To evaluate the qSOFA score for predicting outcomes in dogs in a veterinary ICU and examine whether adding lactate improves its accuracy.

Population:	Canine patients admitted to the Intensive Care Unit (ICU) and transferred to the critical care service at the BluePearl Specialty and Emergency Pet Hospital, New York, USA, between 1 June 2017 and 31 May 2018. Age range was between 2 months and 17 years. 267 dogs (14 septic and 253 non-septic): Septic dogs: 4 septic peritonitis, 3 pneumonia, 3 gastrointestinal translocation, 1 pyometra, 1 urosepsis, 1 bite wound, 1 discospondylitis. Sepsis was defined in this study as (Systemic Inflammatory Response Syndrome (SIRS) plus infection based on cytology, culture or clinical assessment.
Sample size:	267 dogs.
Intervention details:	Based on admission values, patients were assigned a quick Sequential Organ Failure Assessment (qSOFA) score between 0 and 3: respiratory rate (RR) ≥ 22/min = 1, altered mentation (dull, depressed, obtunded, stuporous, minimally responsive, comatose) = 1, systolic blood pressure (SBP) (measured with a Doppler) ≤ 100 mmHg = 1. A score of 0 was assigned if these were within normal limits.
Study design:	Retrospective cohort study.
Outcome Studied:	Survival to discharge and non-survival (death or euthanasia).
Main Findings (relevant to PICO question):	There was no significant difference in qSOFA score when comparing the survivors and non-survivors (P = 0.099). Area Under the Receiver Operating Characteristic (AUROC) curve = 0.6. No significant difference in qSOFA score between surviving septic patients vs non-survivors. Of the dogs with sepsis 10/14 (71.4%) survived and 4/14 (28.6%) died. Overall survival 220/267 (82.4%). 4 patients qSOFA = 0, 168 patients qSOFA = 1, 76 patients qSOFA score = 2, 19 patients qSOFA = 3. Of the non-survivors 38/47 (80.9%) were euthanased and 9/47 (19.1%) died in hospital.
Limitations:	Retrospective study. Few septic patients (14/267) and the survival rate of this group was high (71.4%). Not compared to other illness severity scores (e.g. Sequential Organ Failure Assessment (SOFA), Acute Patient Physiologic and Laboratory Evaluation (APPLE)) to evaluate performance. Euthanised patients were included, not stated whether for financial reasons or poor prognosis. Human cut-off values used for RR and SBP. No control population. No data from the referring hospital; medications or treatments given may have impacted the parameters used for scoring. Panting patients were included. In dogs, this is not necessarily related to a pathology and may have impacted the respiratory rate component of the qSOFA. SIRS criteria used in sepsis definition, and cut-offs used to identify septic dogs were not specified.

**Osgood et al. (2022):** Evaluation of quick sequential organ failure scores in dogs with severe sepsis and septic shock **Aim: **To evaluate the prognostic utility of the qSOFA score in dogs presenting to an emergency service with severe sepsis or septic shock, and to determine its ability to predict the development of severe sepsis and septic shock.

Population:	Canine patients admitted to the Texas A&M University Veterinary Medical Teaching Hospital emergency service, Texas, USA, between 1 January 2010 and 31 December 2019. Digital medical records were reviewed using the search terms “sepsis” or “septic”. Mean age severe sepsis/septic shock (SS/SH) = 7.3 (±3.7) years. Mean age non-infectious SIRS (N-SIRS) = 7 (±4.1) years. 45 dogs; 38 severe sepsis, 7 septic shock. 45 age-matched dogs; non-infectious SIRS.
Sample size:	90 dogs.
Intervention details:	Patients were assigned a quick Sequential Organ Failure Assessment (qSOFA) score between 0 and 3: respiratory rate (RR) > 22/min = 1, altered mentation (dull, obtunded, stuporous) = 1, systolic blood pressure (SBP) (measured with a Doppler) ≤ 100 mmHg = 1. A score of 0 was assigned if these were within normal limits.
Study design:	Retrospective cohort study.
Outcome Studied:	Survival of patient to discharge or non-survival of patient (by death or euthanasia). Created qSOFA30 and qSOFA40 to evaluate different respiratory rate cut-off points: qSOFA30 RR >30 breaths per minute = 1, qSOFA40 = RR >40 breaths per minute = 1, with mentation and blood pressure scores calculated as above.
Main Findings (relevant to PICO question):	The qSOFA score was not predictive of non-survival. Overall survival 45/90 (50%). 29/45 (64.4%) SS/SH, 16/45 (35.6%) N-SIRS. qSOFA30 86/90 (95.6%), qSOFA40 73/90 (81.1%). Of the 29 SS/SH non-survivors, 17/29 (58.6%) were euthanised (15/17 for poor prognosis and 2/17 for financial reasons). Of the 16 N-SIRS non-survivors, 5/16 (31.3%) euthanised due to poor outcome, no financial constraints were mentioned in the files.
Limitations:	Retrospective study. Many patients excluded due to incomplete medical records potentially leading to selection bias. SIRS criteria and qSOFA both use RR so meeting one means likely to meet the other leading to selection bias. Euthanasia included, 2 identified to be financial, others due to perceived poor prognosis. Human cut-off values used for RR and SBP. No healthy control dogs. Combined sepsis and septic shock in one category as too few patients, no additional statistics on these. Using only “sepsis” and “septic” to search records could miss patients that have infectious SIRS due to the different naming system. No data on treatments from the referring hospital. SIRS criteria used in sepsis definition.

**Stastny et al. (2021):** Retrospective evaluation of the prognostic utility of quick sequential organ failure assessment scores in dogs with surgically treated sepsis (2011-2018): 204 cases **Aim: **To evaluate whether admission qSOFA scores predict in-hospital mortality in dogs with surgically treated sepsis.

Population:	Canine patients with a clinical diagnosis of sepsis at the Michigan State University teaching hospital, Michigan, USA, between January 2011 and January 2018. Age range was between 1 month and 15 years. 143 septic peritonitis, 26 septic soft tissue infections, 20 pyometra, 15 pyothorax.
Sample size:	204 dogs.
Intervention details:	A surgical treatment for sepsis was undertaken. Patients were assigned a quick Sequential Organ Failure Assessment (qSOFA) score between 0 and 3: respiratory rate (RR) > 22/min = 1, any abnormal mentation (including dull, obtunded, stuporous) = 1, systolic blood pressure (SBP) (measured with a Doppler) < 100 mmHg = 1. A score of 0 was assigned if these were within normal limits. Patients were further subcategorised into groups: those with a qSOFA score of 1 and those with a qSOFA score of 2 or 3.
Study design:	Retrospective cohort study.
Outcome Studied:	Survival or non-survival of the patient.
Main Findings (relevant to PICO question):	A qSOFA score ≥ 2 was associated with mortality (P < 0.0001; OR 7.1 95% CI 6.1–34.3). qSOFA scores were significantly different between survivors and non-survivors (P < 0.0001). Discrimination between survivors and non-survivors based on qSOFA was good (Area Under the Receiver Operating Characteristic (AUROC) curve = 0.81 95% CI 0.75–0.87). Overall survival 141/204 (69%). There were 63/204 (30.9%) patients in the qSOFA ≥ 2 group and 43/63 (68.3%) were non-survivors.
Limitations:	Retrospective study. 143/204 (70.1%) of the dogs had septic peritonitis which may have resulted in a population bias. 1105/1309 records did not meet the study criteria. Excluding dogs with milder signs and no cultures may have selected for higher qSOFA scores and contributed to a population bias. Excluding dogs that were euthanised without treatment may have artificially decreased the mortality rate. Including euthanasia without determining the cause could have confounded the results. Human cut-off values used for RR and SBP. No control population. Not compared to other illness severity scores (eg Sequential Organ Failure Assessment (SOFA), Acute Patient Physiologic and Laboratory Evaluation (APPLE)) to evaluate performance. No information on treatments from the referring hospital. Systemic Inflammatory Response Syndrome (SIRS) criteria used in sepsis definition.

## Appraisal, application and reflection

The quick Sequential Organ Failure Assessment (qSOFA) serves as a prognostic bedside test designed to facilitate early intervention for patients at risk of deterioration (Evans et al., 2021). It is less robust than the full Sequential Organ Failure Assessment (SOFA) score which evaluates six organ systems using respiration (PaO2/FiO2), platelet count, bilirubin concentration, blood pressure, abnormal mentation, and creatinine concentration (Ripanti et al., 2012). Though SOFA has demonstrated reasonable prognostic capabilities in dogs (Ripanti et al., 2012; Kalogianni et al., 2022), it requires laboratory data, which can result in delays to treatment. In addition, while the SOFA score focuses on the clinical criteria that demonstrate organ dysfunction to assess illness severity (Singer & Shankar-Hari, 2018), qSOFA is designed to quickly identify patients at risk of poor outcomes. Its primary benefit is in rapid bedside identification, particularly in resource-poor settings where laboratory data is not readily available (Singer et al., 2016).

Unlike the Acute Patient Physiologic and Laboratory Evaluation (APPLE) and its abbreviated version, the APPLEfast, qSOFA has not been validated in dogs (Hayes et al., 2010a). APPLE was evaluated in a prospective study and was able to discriminate between survivors and non-survivors with high predictive power (Giunti et al., 2014). As diagnosis independent scores they have a wide applicability, however both APPLE and APPLEfast require laboratory data which is their limitation in terms of speed and ease of use (Hayes et al., 2010a). As such, qSOFA is one of the few available scoring systems that does not require laboratory testing and has the potential to influence treatment rapidly at the bedside.

The transferability of a model depends on the similarities between the population used in its construction and the end-user population (Hayes et al., 2010b). The fact that qSOFA must be adjusted for human paediatrics (Goldstein et al., 2005; Schlapbach et al., 2017), draws into question whether it could possibly retain predictive accuracy in veterinary medicine without altering its cut-off points to account for the physiological variation from the adult human model.

The normal respiration rate for a dog ranges from 20–30 breaths per minute, and respiratory rate can also be elevated for physiological reasons such as panting for thermoregulation (Englar, 2019). Given the range of respiration rates that can be considered normal in dogs, a cut-off point of 22 or more is not an ideal threshold for identifying at-risk patients in veterinary practice.

Hospitalisation stress in dogs is often unavoidable, and it is necessary for the clinician to distinguish psychogenic stress from the physiologic stress that arises from systemic illness, trauma or surgery (Hekman et al., 2014). Stress can influence respiratory rate for non-pathological reasons (Englar, 2019) and as such, measurements should be interpreted with the patient’s affective state in mind.

All four studies used the human cut-off points of more than 22 breaths per minute and systolic blood pressure of less than or equal to 100 mmHg. Osgood et al. (2022) tested higher cut-off points for respiration rate to account for panting, however it was applied generally to the study population without further stratification. Ortolani & Bellis (2021) and Donati et al. (2022) did not consider panting in their study design, but Stastny et al. (2021) excluded dogs whose respiration rate was recorded as panting. None of the studies considered the impacts of fear and stress in their study designs, though Osgood et al. (2022) noted that in a prospective study setting, measurements could be taken after addressing stress and anxiety to improve accuracy. Donati et al. (2022) also noted that the use of human cut-offs was a limitation of the application of qSOFA in canines and recommended that further studies be undertaken to determine species-specific parameters. Adjusting the qSOFA parameters would improve its relevance in veterinary practice. However, doing so would require substantial validation work to ensure consistency across diverse canine populations, potentially complicating its simplicity.

All four studies used the Area Under the Receiver Operating Characteristic (AUROC) curve to represent the ability of qSOFA to distinguish between survivors and non-survivors. However, the presentation may result in difficulty for clinicians to interpret and apply the results. Stastny et al. (2021) and Donati et al. (2022) both used odds ratios to represent the likelihood of mortality for a qSOFA score less than or equal to 2, but only Donati et al. (2022) and Ortolani & Bellis (2021) provided sensitivity and specificity information to qualify the AUROC. Table 1 highlights the variability in qSOFA’s performance across studies. Notably, the AUROC ranged from 0.51 to 0.81, indicating inconsistent discriminatory ability, while sensitivity and specificity (where reported) varied widely, further complicating clinical interpretation.

**Table 1:** Summary of reported results.

Study	AUROC	OR	Sensitivity %	Specificity %
Ortolani & Bellis (2021)	0.6	-	44*	34*
Stastny et al. (2021)	0.81	7.1	-	-
Donati et al. (2022)	0.72	6.51	77.8	66.7
Osgood et al. (2022)	0.51	-	-	-

AUROC = Area Under the Receiver Operating Characteristic curve, OR = odds ratio.*From graphed results.

When considering the results from Stastny et al. (2021), though we can assume that the AUROC of 0.81 was selected to optimise both sensitivity and specificity we cannot know whether one was compromised in favour of the other. This is important for a prognostic tool as a poorly sensitive test cannot be used as a prognostic indicator. This would result in a high false negative rate and missed cases. It is also important to know the extent to which specificity has been compromised, given that low specificity would result in a higher false positive rate and a falsely poor prognosis which, in veterinary medicine, could influence owners towards euthanasia unnecessarily. The balance between these metrics is crucial in determining whether qSOFA can reliably guide clinical decisions.

Risk factors for euthanasia are often client factors, such as financial constraints or caregiver burden which are not necessarily related to prognosis (Pegram et al., 2021). This influence, along with the client’s subjective assessment of the animal's suffering, complicate the evaluation of euthanasia bias in prognostic scoring (Hayes et al., 2010b). Additionally, if a clinician perceives certain clinical signs to be negative, this will be reflected in their recommendations, creating a mortality association regardless of whether one truly exists (Hayes et al., 2010a). However, whilst discrimination of models can be improved by removing euthanasia cases, this often greatly limits and biases the data (Hayes et al., 2010a; Hayes et al., 2010b).

Ortolani & Bellis (2021) did not remove euthanasia from their data which may have biased the outcome. Stastny et al., (2021) and Osgood et al. (2022) did not include euthanasia before treatment, however euthanasia after treatment was still included. Though, in the case of Osgood et al. (2022), only two of the included patients were identified as being euthanised for financial reasons. Only the study by Donati et al. (2022) removed economic euthanasia or euthanasia for unknown reasons from their data, including only mortality from natural causes and euthanasia due to disease progression or lack of response to treatment. Given the retrospective design of these studies, there is also a possibility that the reasons for euthanasia may not have been accurately captured in the patient files.

Referral data would aid in contextualisation and interpretation of the score. While qSOFA is designed to assess patients at any stage of hospitalisation, documenting referral timing and any treatments given prior to referral may provide additional context. The components of qSOFA (respiratory rate, blood pressure, and mentation) can all be artificially lowered by common veterinary sedation and analgesic agents (Nishimura et al., 2018; Englar, 2019). As such, drug-induced alterations in mental state and other physiological effects must also be taken into consideration when calculating a qSOFA score.

All four studies used admission data which should have avoided the effects of analgesia or sedation, however as these studies were conducted in referral hospitals, this would not account for medications given by the referring clinics. None of the studies noted any data from the referring facilities, though Ortolani & Bellis (2021) considered this limitation in their discussion and Donati et al. (2022) maintained that no fluids or supportive therapies had been given prior to referral.

Prognostic studies, such as for illness severity score evaluation, appraise clinical information and patient variables to discern the predictors of a given outcome (Ono et al., 2013). The highest quality evidence is derived from an adequately powered, prospective, blinded cohort study (Burns et al., 2011). Retrospective cohort studies, such as the ones appraised in this Knowledge Summary, comprise a lower level of evidence (Burns et al., 2011). Additionally, qSOFA includes mentation, a subjective measure, that may differ between clinicians. If recorded accurately and objectively, this data can be reliable. However, retrospective studies depend on clinical notes that were not specifically recorded for research purposes, and inconsistencies in documentation can impact interpretation and objectivity (Talari & Goyal, 2020).

All four studies were retrospective and suffered the same limitations of this study design. They all excluded records with missing values as these data were required to complete the qSOFA calculation. However, this could have resulted in selection bias. All noted the number of exclusions and the reasoning, though only Stastny et al. (2021) listed it as a limitation. However, the study by Osgood et al. (2022) did include a control group to combat the effect of different baseline characteristics.

The external validity of qSOFA in veterinary medicine remains uncertain. While qSOFA was designed to identify patients at risk of poor outcomes regardless of underlying illness (Singer et al. 2016), its application to septic and non-septic canine populations has yielded mixed results. This raises the question of qSOFA’s clinical utility and whether performance varies based on case selection. Including broader populations may dilute the specificity of findings and reduce clinical applicability. However, given that non-septic conditions can also lead to critical deterioration, evaluating qSOFA beyond sepsis remains relevant. At present, it is unclear whether the findings of these studies can be generalised to other veterinary settings.

The definition of sepsis must also be carefully considered. In the evaluated papers, sepsis was defined as either Systemic Inflammatory Response Syndrome (SIRS) plus a documented or suspected infection or as SIRS plus infection along with evidence of organ dysfunction. These differing definitions could impact the performance of the score as they reflect varying degrees of illness severity. Furthermore, the Sepsis-3 guidelines found that the SIRS criteria had limited diagnostic utility for sepsis as they were more reflective of an inflammatory response than a dysregulated host response, leading to excessive false positives and insufficient discrimination (Singer et al. 2016).

Two of the evaluated papers used SIRS as part of their inclusion criteria (Stastny et al. 2021; Osgood et al. 2022), and Ortolani & Bellis (2021) used SIRS to identify septic cases in their patient population. Although SIRS criteria were employed by Donati et al. (2022), they were adjusted for dogs using proposed increased heart and respiratory rate values and were used for comparison with qSOFA as opposed to inclusion criteria for the study.

Though the SOFA score has been shown to have good prognostic capabilities in dogs (Ripanti et al., 2012; Kalogianni et al., 2022), none of the studies evaluated in this Knowledge Summary compared its efficacy with that of qSOFA.

Given that APPLE and APPLEfast are the only validated scoring systems for dogs, it would also be useful to assess how they compare to qSOFA. None of the studies compared qSOFA with APPLE, though Osgood et al. (2022) utilised APPLEfast, finding it to be significantly associated with mortality, unlike qSOFA in that study. Both Ortolani & Bellis (2021) and Stastny et al. (2021) noted the lack of comparison to other scoring systems as a limitation of their studies.

The reviewed evidence can neither prove nor disprove the utility of qSOFA as an assessment or prognostic tool for septic canine patients. While this means that qSOFA is not yet suitable for routine clinical use in veterinary hospitals, the potential remains for it to be an instrument of rapid identification to prompt clinicians to escalate therapy or increase monitoring frequency, similar to its use in human medicine (Singer et al., 2016). There may also be a use for it in general practice to guide referral decisions by identifying patients that are likely to have poor outcomes or require more interventions. It is, however, not suitable for informing clients about the prognosis of an individual dog, nor was it intended for that purpose.

When surveyed, clinicians rank ease of use above the accuracy and objectivity of a scoring system (Hayes et al., 2010a). Consequently, the greatest barriers to adoption are score complexity and inconvenience (Hayes et al., 2010b). Here the qSOFA is at an advantage, though its lack of validation in a veterinary context remains a drawback. The strength of evidence is not such that it can support a change in practice, however, the qSOFA may still guide patient management if the clinician is aware of its limitations. Clinicians regularly outperform scoring models in predicting mortality in individual patients, and combining a scoring tool with a clinician’s assessment can further improve prediction accuracy (Hayes et al., 2010b). Additionally, given that the barriers to adoption relate to ease of use, it is arguably more useful to have a score that is less robust but quicker and cheaper to apply than to have a more robust scoring system that results in extra costs and delays to treatment.

The strength of evidence regarding the efficacy of qSOFA is weak and the findings of the four studies yield conflicting results. Ortolani & Bellis (2021) and Osgood et al. (2022) suggest that qSOFA cannot reliably distinguish between survivors and non-survivors, and in contrast, Stastny et al. (2021) and Donati et al. (2022) demonstrate significant differences in qSOFA scores between the two groups. This disparity highlights the need for further investigation to clarify qSOFA’s prognostic value in septic canine patients.

All four studies were retrospective and thus weakened by the limitations of that design. Additionally, without a validated reference for the number of breaths per minute indicating respiratory dysfunction in dogs of different sizes, the qSOFA cannot be effectively applied. Future research should focus on validating qSOFA using canine parameters.

When qSOFA was first proposed, the sepsis task force strongly advocated for prospective validation in different health care settings to confirm its robustness (Singer et al., 2016). A future, prospective, blinded cohort study should be conducted to compare the efficacy of qSOFA with APPLEfast for predicting mortality in dogs with sepsis. This study would ideally control for confounding factors such as hospital-induced stress and euthanasia and adjust physiological parameters for canines. This approach would address the variability in canine physiology that may affect the prognostic capabilities of qSOFA. It would also assess its performance against a validated veterinary illness severity score to identify its relative strengths and limitations.

## Methodology

**Search Strategy:** 

Databases searched and dates covered:	CAB Abstracts via Web of Science 1910 to 10 January 2025 PubMed via NCBI 1946 to 10 January 2025 Scopus via Elsevier 1970 to 10 January 2025
Search strategy:	CAB Abstracts: TS=((dog OR dogs OR canine OR canines) AND (qSOFA OR "quick sequential organ failure assessment") AND (mortality OR survival OR prognosis) ) PubMed: (dog OR dogs OR canine OR canines) AND (qSOFA OR "quick sequential organ failure assessment") AND (mortality OR survival OR prognosis) dogs [MeSH] Scopus: ( TITLE-ABS-KEY ( ( dog OR dogs OR canine OR canines ) ) AND TITLE-ABS-KEY ( ( qsofa OR "quick sequential organ failure assessment" ) ) AND TITLE-ABS-KEY ( ( mortality OR survival OR prognosis ) ) )
Dates searches performed:	10 Jan 2025

**Exclusion / Inclusion Criteria:** 

Exclusion:	Not related to the PICO parameters. Single case reports. Conference proceedings. Book chapters. Systematic reviews or meta-analyses. Papers older than 20 years.
Inclusion:	Canine patients. qSOFA score evaluated for prognostic efficacy. Veterinary hospital setting.

**Search Outcome:** 

Database	Number of results	Excluded – not canines	Excluded – different topic	Total relevant papers
CAB Abstracts	4	0	0	4
PubMed	4	0	0	4
Scopus	6	1	1	4
Total relevant papers when duplicates removed				4

## Acknowledgements

I would like to thank Dr Samantha Livingstone for providing academic guidance.

## ORCiD

Jacqueline Chappell: https://orcid.org/0009-0004-6496-165X

## Conflict of Interest

The author declares no conflicts of interest.
